# Tel1^ATM^ dictates the replication timing of short yeast telomeres

**DOI:** 10.15252/embr.201439242

**Published:** 2014-08-13

**Authors:** Carol Cooley, Anoushka Davé, Mansi Garg, Alessandro Bianchi

**Affiliations:** Genome Damage and Stability Centre, School of Life Sciences, University of SussexBrighton, UK

**Keywords:** DNA replication, origin firing, replication timing, Tel1, telomeres

## Abstract

Telomerase action is temporally linked to DNA replication. Although yeast telomeres are normally late replicating, telomere shortening leads to early firing of subtelomeric DNA replication origins. We show that double-strand breaks flanked by short telomeric arrays cause origin firing early in S phase at late-replicating loci and that this effect on origin firing time is dependent on the Tel1^ATM^ checkpoint kinase. The effect of Tel1^ATM^ on telomere replication timing extends to endogenous telomeres and is stronger than that elicited by Rif1 loss. These results establish that Tel1^ATM^ specifies not only the extent but also the timing of telomerase recruitment.

## Introduction

The preservation of genome integrity in eukaryotes requires protective nucleoprotein structures at chromosome ends, the telomeres, which are maintained by telomerase, a reverse transcriptase-like ribonucleoprotein responsible for the synthesis of the telomeric DNA repeats. Telomerase acts preferentially at the shortest telomeres, which in yeast are marked for elongation by association with the Tel1^ATM^ kinase [1–3]. Telomerase action is coordinated with conventional DNA replication of the bulk of the telomere [4] taking place from replication forks originating from subtelomeric origins of DNA replication [5,6]. Initiation of DNA replication from a DNA-bound pre-replicative complex made of the origin recognition complex (ORC) and the origin-unwinding hexameric MCM helicase requires CDK and DDK kinase action to promote MCM activation and recruitment of additional factors for replication fork assembly, including the DNA polymerases. In eukaryotic genomes, these events do not take place simultaneously at all origins but follow a controlled programme. In budding yeast, origins (or autonomously replicating sequences—ARSs) have defined DNA sequence requirements, but their activity and timing is affected by chromatin context and histone modifications [7–9].

Although yeast telomeres are among the latest-replicating regions in the genome, this replication pattern is dependent on telomere length and telomeres in the shorter length range are replicated by early-firing subtelomeric origins [10,11]. Even though it has been shown that both Rif1 and the yeast Ku protein (Yku) are required for the late replication of yeast telomeres [11,12], it remains unclear how telomere length acts as a determinant of the timing of origin firing.

## Results and Discussion

### Induction of a DSB flanked by short telomeric tracts leads to a change in the timing of origin firing at the broken locus

**Figure 1:**
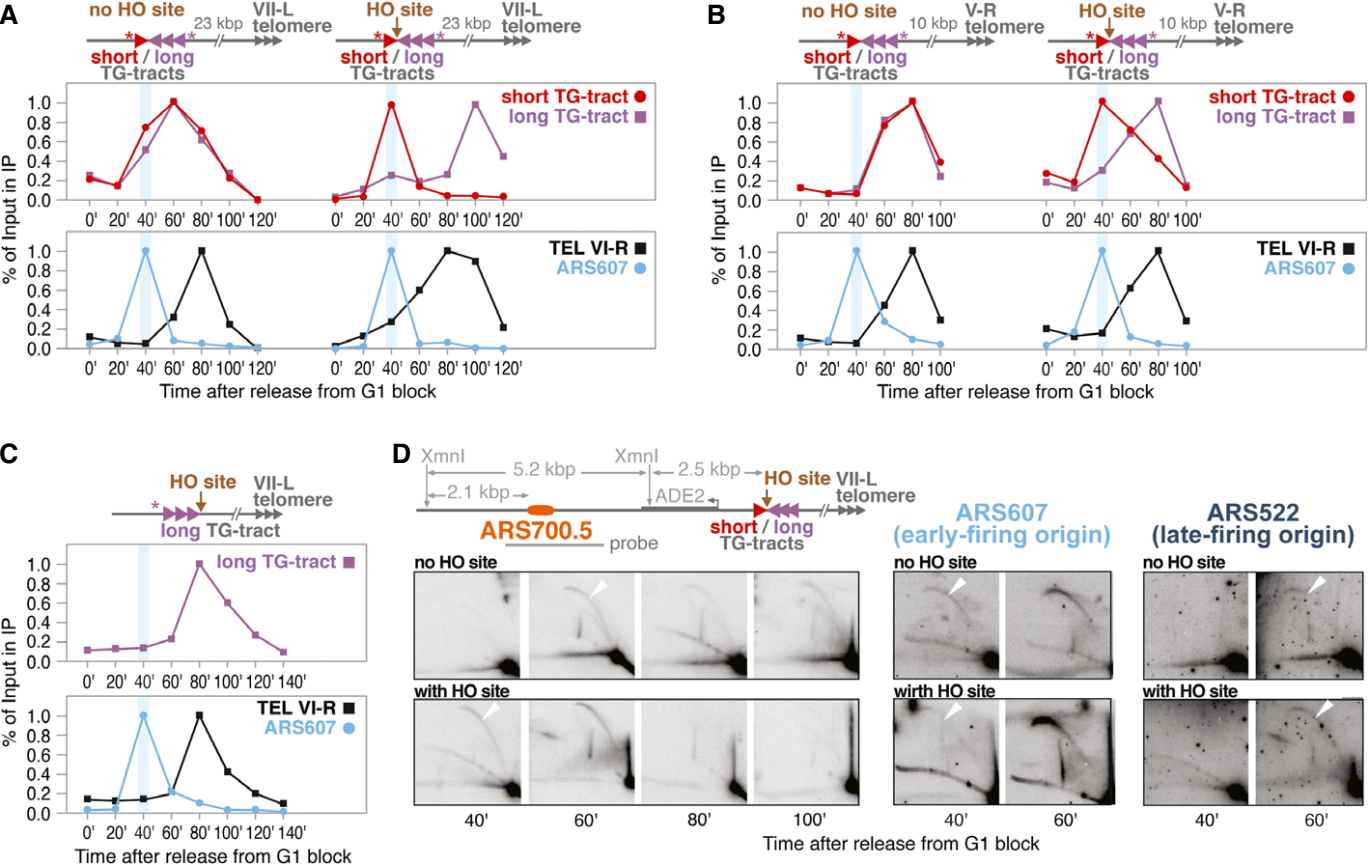
Induction of a DSB flanked by short telomeric tracts leads to early S-phase origin firing at the break site A DSB was introduced at a TG80-HO-CA250 cassette at the *adh4* locus by induction of the HO endonuclease with galactose during G1 block with α-factor (right). A control strain lacked the HO site (left). Samples were collected at the indicated times after release at 18°C and subjected to ChIP analysis of Polε-13Myc association with the indicated loci. Two loci were included in the analysis as early and late S-phase markers: *ARS607* and telomere *VI-R*, respectively. In all figures, asterisks indicate the positions of PCR amplicons used for qPCR.Same as in (A) but the TG80-HO-CA250 cassette was inserted at the subtelomeric *YER188W* locus at chromosome *V-R*.Same as in (A) but a TG250-HO cassette was used.Samples from synchronised cultures were collected at the indicated time points, cells were killed with sodium azide and DNA was processed and probed for two-dimensional electrophoresis analysis of replication intermediates. The ‘bubble’ arc, indicative of a replication bubble arising from origin firing, is shown by white triangles at its earliest time of detection.

To test whether the replication timing of short telomeres might be related to their transient uncapping [1–3,13], we investigated the behaviour of a double-strand break (DSB) generated in G1 at a late-replicating locus. The DSB was flanked by a short array of telomeric repeats (short-TG hereafter; bearing about 80 bp of yeast telomeric sequences). A long TG-tract was present at the distal end of the break, since a large number of telomeric repeats were required to make the locus late replicating in the absence of cleavage (Supplementary Fig S1A and B). Each array was arranged in the telomere-like orientation towards the free end. We used chromatin immunoprecipitation (ChIP) to assess association of the DSB with the leading-strand DNA polymerase Polε (coded by the *POL2* gene), which binds to activated origins and travels with the replication fork. Whereas in the uncut locus Polε association at the short TG-tract peaked at 60 min after release (Fig 1A, left), cells that received a DSB displayed a peak at 40 min (Fig 1A, right), indicating that the DSB had caused a shift in the timing of the association of the polymerase with the locus (the 40-min time point, highlighted with a blue bar in all ChIP figures, is indicative of early S phase and coincides with peak binding of the early-firing origin *ARS607*). When we inserted the TG80-HO-CA250 cassette at a second subtelomeric site, on chromosome *V-R*, this locus too displayed late S-phase association with Polε when uncleaved (Fig 1B, left), and a peak of Polε association in early S phase upon DSB formation (Fig 1B, right). Importantly, the shift in Polε binding at the DSB was not observed with the long array (Fig 1C and Supplementary Fig S1B), or at the distal end, which bears the long TG-tract array (Fig 1A and B, right). These results demonstrate that the introduction of a DSB near short, but not long, arrays of telomeric repeats changes the timing of association of the leading DNA polymerase from late to early S phase.

To demonstrate directly that Polε association with the DSB was related to origin activation, we analysed replication intermediates by two-dimensional gel electrophoresis. To address the activity of *ARS700.5*, an origin located in the vicinity of the *ADH4* locus [10,14], we analysed this region by 2D gels before and after short-TG DSB formation. In agreement with the ChIP data, in the absence of DSB, we detected a ‘bubble arc’, consistent with origin firing at *ARS700.5*, late in S phase (Fig 1D, left, top panels), similar to late origin *ARS522* (Fig 1D, right). Strikingly, DSB induction led to the appearance of the arc 20 min earlier (Fig 1D, left, bottom panels) and coincident with its appearance at the early origin *ARS607* (Fig 1D, middle). The DSB did not affect the timing of the two control origins. These results indicate that recruitment of Polε to the DSB is related to origin firing and that break formation specifically affects the replication programme of the affected locus. The conclusion is further supported by analysis of Cdc45 binding to the break site (Supplementary Fig S1C). Thus, similarly to short telomeres, DSBs flanked by short TG-tracts led to a change in the activation time of origins of DNA replication from late to early S phase.

In the distal fragment severed by the DSB at *VII-L*, a (likely unique) replication origin is located in the subtelomeric X element. Interestingly, a construct with a TG250-HO-CA80 cassette did not lead to early replication at either DSB end (Supplementary Fig S1D), suggesting that the endogenous telomere in this fragment (which is much closer to the origin than the DSB end is) might exert a repressive effect.

### The shift in replication timing at a DSB flanked by short telomeric arrays requires Tel1^ATM^

**Figure 2:**
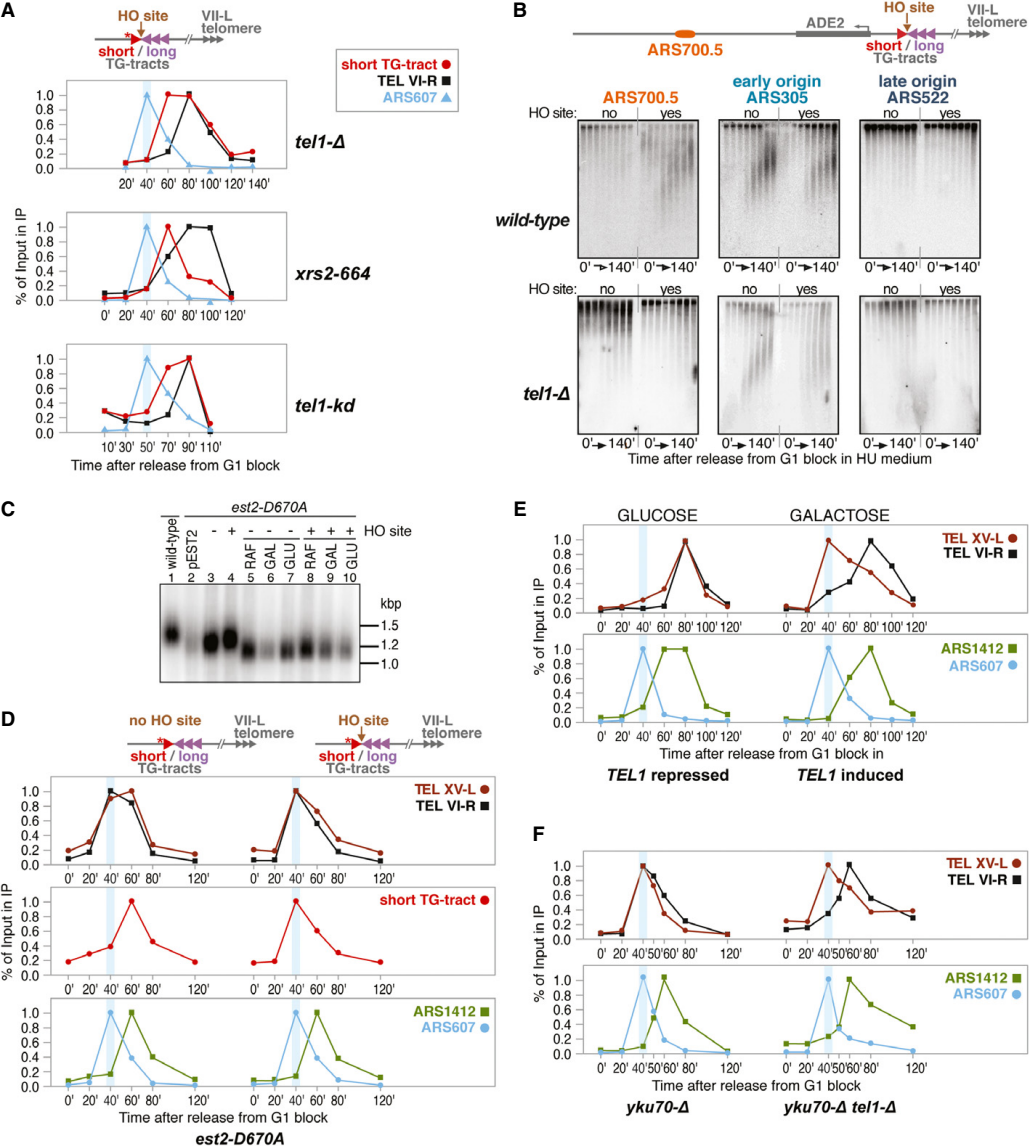
Requirement of Tel1 for early S-phase origin firing at a DSB flanked by short telomeric arrays and at short telomeres Strain as in Fig 1A, right, but carrying a *tel1*Δ (top), *xrs2-664* (lacking the last 190 amino acids; middle) or *tel1-kd* (kinase-dead; bottom) were analysed for Polε-13Myc association as in Fig 1.Analysis of replication intermediates by alkaline gel electrophoresis. Cells were arrested in G1 in galactose as in Fig 1 and released into medium containing 200 mM HU. Replication intermediates were separated on alkaline gels and detected by hybridisation with probes against the indicated origins.Southern blot showing telomere lengths of strains analysed in (D). Lane 1, wt; lane 2, *est2-D670A* with *EST2* complementing plasmid; lanes 3 and 4, *est2-D670A* strain (without and with HO site, respectively) immediately after plasmid loss; lanes 5–7, same strain as in lane 3 but after over-night growth in raffinose before (lane 5) and after (lane 6) incubation in galactose, and at end of 2-h incubation in glucose (lane 7); lanes 8–10, same as lanes 5–7 but for same strain as in lane 4. Plasmid loss was achieved on 5-FOA plates to select against the plasmid-borne *URA3* marker.ChIP analysis of Polε association of *est2-D670A* strains analysed in (C). Time point 0′ corresponds to samples 6 and 9 in (C), and time point 120' to samples 7 and 10. The *V-R* and *XV-L* telomeres are *X*-only telomeres (no *Y*′ elements).ChIP analysis of Polε association in a strain where the *TEL1* gene was under control of a galactose-inducible promoter. Cells were either arrested in glucose- (left) or galactose-containing medium (right) in the presence of α-factor and released in glucose medium.Strains carrying a deletion of *YKU70* were analysed for Polε-13Myc association as in Fig 1. The strain on the right also carries a deletion of *TEL1*.

Because short-TG DSBs, like short telomeres and unlike long-TG DSBs, recruit high levels of Tel1 [1,2,10,15], we sought to determine whether the kinase might be required for the change in the origin firing programme at the short-TG DSB. When we monitored Polε recruitment at this locus in a strain lacking Tel1, we failed to observe the early S phase peak of association with the proximal end of the break (Fig 2A, top). We similarly could not detect a significant level of Polε association in early S at this locus in cells lacking the C-terminal domain of Xrs2 (Fig 2A, middle), which is required for Tel1 localisation to DSBs and telomeres [1]. Finally, the kinase activity of Tel1 was required for the shift in timing of Polε binding (Fig 2A, bottom).

Consistent with the above results, alkaline gel analysis of the replication intermediates obtained from cells released into hydroxyurea (HU), which specifically suppresses late origins, detected firing at *ARS700.5* in the presence of a DSB, but not in its absence (Fig 2B, top left). Early and late origins *ARS305* and *ARS522,* respectively, served as controls (Fig 2B, middle and right). A strain lacking Tel1, on the other hand, failed to produce replication intermediates at *ARS700.5* even in the presence of cleavage (Fig 2B, bottom left). Taken together, these results reveal that the kinase activity of Tel1 is required for the early S-phase activation of an origin of DNA replication at a DSB flanked by short arrays of telomeric repeats.

### The early replication of short yeast telomeres requires Tel1^ATM^

To generate yeast cells with short unmodified telomeres, we used a catalytically inactive telomerase (Est2) allele. Telomeres in cells bearing this allele will progressively shorten and were therefore maintained via an Est2 plasmid that was ejected from the cells before analysis, at which time telomere length was about 160 bp (Fig 2C, compare lanes 5–10 to lane 1). ChIP analysis of Polε telomere binding in these cells indicated that both the short endogenous *VI-R* and *XV-L* telomeres replicated early in S phase (Fig 2D, top). Polε recruitment at the uncut *adh4* locus was not prominent in early S and, as expected, became so upon DSB formation (Fig 2D, middle). These results indicate that early replication timing is a general feature of short yeast telomeres and that the early recruitment of Polε is independent of TG-strand synthesis, both at endogenous telomeres and the short-TG DSB.

Notably, although the MRX complex and Tel1 (which act in a single pathway to regulate telomerase action) have very short telomeres [16–18], cells lacking any of these components do not replicate their short telomeres early (Fig 2A) [19], consistent with our data that Tel1 activity is required for their early replication. To further address this role of Tel1 at endogenous telomeres, we used an inducible form of Tel1, which is repressed in glucose medium and induced by galactose [13]: under conditions of Tel1 repression, Polε telomere association took place in late S (Fig 2E, left), whereas exposure to galactose medium during the G1 arrest dramatically changed the profile of Polε association with the *XV-L* telomere, shifting it to early S (Fig 2E, right). Interestingly, telomere *VI-R* was largely refractory to early S-phase association after galactose induction within this first cell cycle, suggesting that telomere-specific effects are also at play.

**Figure 3:**
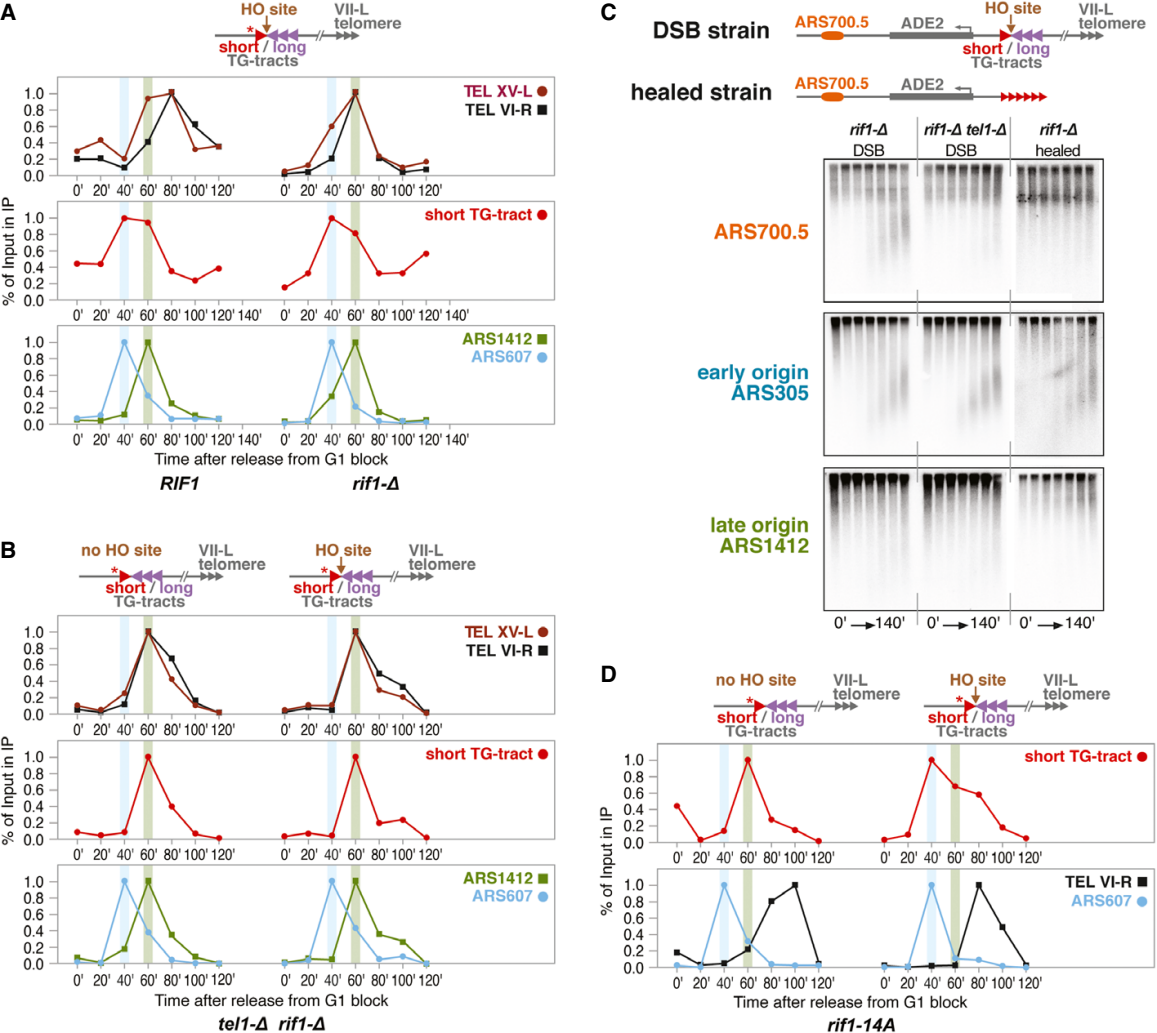
Telomere shortening and loss of Rif1 affect replication timing independently ChIP analysis of Polε association with indicated loci in *rif1*Δ (left) and *rif1*Δ *tel1*Δ (right) cells during the cell cycle.A *rif1*Δ *tel1*Δ strain carrying cassettes at *adh4* for HO cleavage (right), or not (left), was processed as described as in Fig 1A.Strains of the indicated genotypes were processed as in Fig 2B. The ‘healed’ strain was obtained by inducing cleavage at the TG80-HO-TG250 cassette and allowing a new telomere to stabilise.A strain carrying alanine substitutions at all Ser and Thr residues within the 14 SQ/TQ sites within Rif1 was analysed as in Fig 1A.

The short telomeres of cells lacking Yku replicate in early S [11,12,20], raising the possibility that this might be due to their uncapped state and documented increased Tel1 recruitment [2]. In agreement with this idea, while Polε, as expected, peaked in early S at telomeres in *yku* cells (Fig 2F, left; compare to wild-type in Fig 3A), it associated later in S phase at the *VI-R* telomere if Tel1 was also absent (Fig 2F, right). The suppression of the early replication timing of telomere *XV-L*, which is one of the earliest replicating telomeres, was only minor in the double mutant, again pointing to telomere-specific effects. Taken together, these results suggest that Tel1 is required for the early replication of short budding yeast telomeres.

### Tel1 acts independently of Rif1 in affecting the replication timing of telomeres

Telomere-bound Rif1 determines the replication timing of budding and fission yeast telomeres [11,21] by recruiting protein phosphatase 1 to reverse the action of DDK on the MCM helicase [22–24]. Because Rif1 is an *in vivo* substrate of the ATM/ATR kinases, in principle Tel1 could act by repressing the origin-suppressing activity of Rif1 at telomeres. However, this view is not supported by a comparison of the replication timing of short and *rif1* telomeres: whereas short telomeres and short-TG DSBs displayed early S Polε binding (Figs 1, 2, 3, blue bars), in *rif1* null mutants, we observed an anticipation of only about 20 min of Polε telomere association, to coincide with the late-replicating *ARS1412* (Fig 3A, top and bottom panels, green bars) [22]. The fact that the telomere association of Polε in *rif1* cells, although earlier than in wild-type cells, was not as early as that seen at short telomeres suggests that Rif1 cannot be the sole (or main) target of Tel1 action at short telomeres. Epistasis analysis further supports this interpretation since Tel1 was still required for the early replication of the short-TG DSB in cells lacking Rif1 (Fig 3B, middle). This observation is particularly significant, since it eliminates the possibility that in *rif1* cells the endogenous telomeres (which are long) might achieve only a partial shift in replication timing due to their length: at the DSB, the TG-tract is very short but, in the absence of Tel1, association of Polε remains late regardless of the presence of Rif1 (it coincides with late *ARS1412*, rather than early *ARS670*, Fig 3B, middle).

These findings were further confirmed by an analysis of replication intermediates in cultures arrested in HU. Consistent with the ChIP data, no replication intermediates were observed at *ARS700.5* at the short-TG DSB in *rif1 tel1* cells (Fig 3C, top centre). In addition, in a *rif1* strain where a new telomere had been allowed to form and stabilise at the DSB, no replication intermediates were detected (Fig 3C, top right), in agreement with the idea that loss of Rif1 only lends a relatively minor reprieve to the late replication programme of yeast telomeres.

Taken together, these results do not support a simple model for a role of Tel1 upstream of Rif1 in regulating replication timing. To directly test the hypothesis that Rif1 might be a target of Tel1 in this pathway, we created strains bearing an allele of Rif1 with all 14 serines or threonines in the Mec1^ATR^/Tel1^ATM^ consensus sites mutated to alanines (*rif1-14A*). In this mutant strain, the pattern of Polε recruitment to telomeres and to short-TG DSBs was unaltered compared to wild type (Fig 3D), suggesting that putative phosphorylation of Rif1 by Tel1 is not sufficient to relieve the origin-delaying action of Rif1.

### Tel1 is needed for the G1 phase association of Cdc45 with an origin located near the short TG-tract DSB

**Figure 4:**
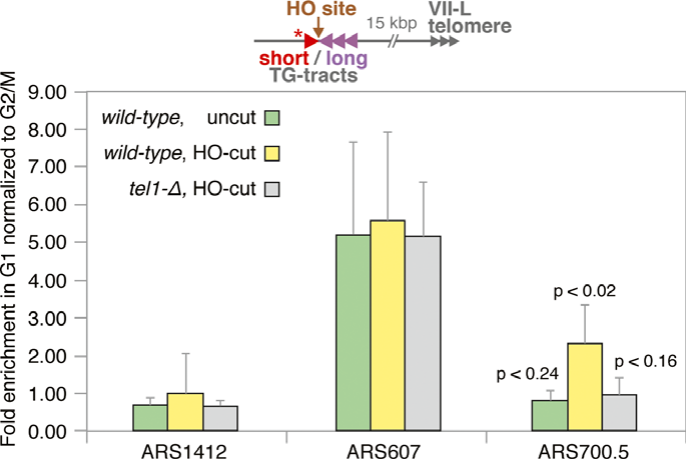
Tel1 is required for loading of Cdc45 at an origin proximal to a DSB flanked by short telomeric arrays ChIP analysis of Cdc45 association with *ARS700.5* (see Fig 1C) and with early- and late-firing (*ARS607* and *ARS1412*, respectively) origins in G1. Cells were arrested in G2 with nocodazole and then released into galactose-containing medium with α-factor for 4 h. Samples were collected for ChIP analysis at the end of the nocodazole and α-factor incubations. Averages of at least five samples for each strain/conditions from two independent experiments are shown. Error bars represent standard deviations. The significance of the increase in G1 signal over G2/M for *ARS700.5* is indicated (*P*-values were calculated by exact binomial test).

Because the DDK-dependent binding of Cdc45 and Sld3 in G1 is a characteristic of early origins [25], we decided to test whether Tel1 plays a role in assisting the loading of Cdc45 at an origin flanking the short-TG DSB. For this purpose, we arrested cells in the M phase with nocodazole and then released them into medium containing α-factor and galactose, to prevent exit from G1 while inducing a short-TG DSB in the proximity of *ARS700.5*. As expected, we detected robust enrichment of Cdc45 in G1, compared to M phase, at the early *ARS607*, but not at the late *ARS1412* (Fig 4, centre and left, respectively). We also failed to observe G1 enrichment for Cdc45 at *ARS700.5* in a strain lacking an HO site at this locus (Fig 4, right, green bar); in contrast, introduction of the short-TG DSB at *adh4* gave rise to an increase in Cdc45 G1 phase binding at this origin (Fig 4, right, yellow bar). Strikingly, the G1 enrichment in Cdc45 binding at the DSB-flanking origin was lost in the absence of Tel1 (Fig 4, right, grey bar). These results suggest that Tel1 acts to set the firing time of origins by affecting the loading of Cdc45 in the G1 phase. One possibility is that Tel1 acts directly on the MCM helicase to promote Cdc45 recruitment, in a manner analogous to DDK. Interestingly, the association of DDK with MCMs requires priming phosphorylation events, some of which are the result of Mec1^ATR^ action [26]. A similar mode of action for Tel1 at short telomeres is not currently supported by our analysis of *mcm4* and *mcm6* mutants lacking the Mec1 sites at their N-termini (Supplementary Fig S2). It is, however, possible that other, possibly redundant, targets of Tel1 exist within the MCM complex.

### Requirement of telomeric repeats for replication timing shift at a DSB

**Figure 5:**
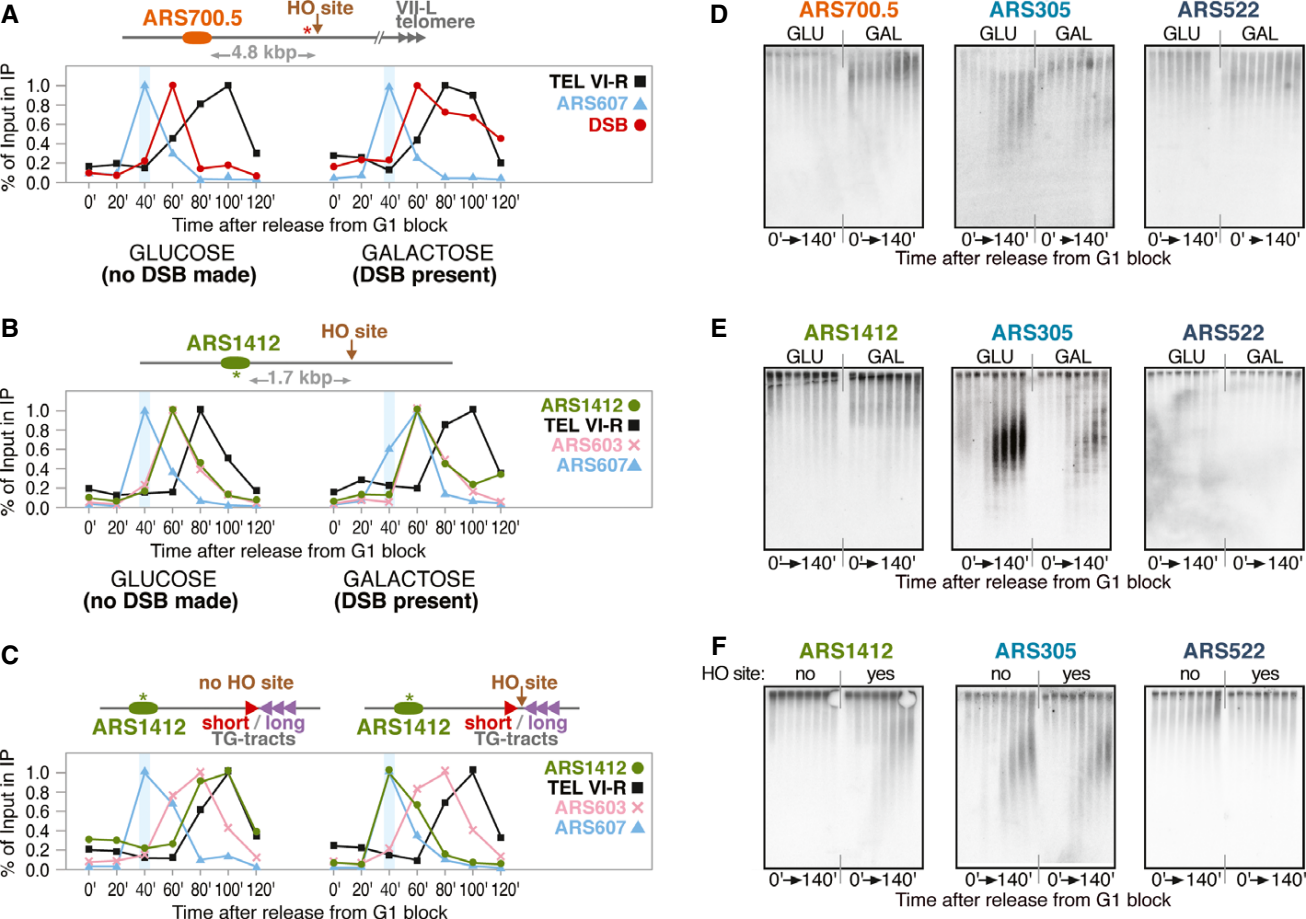
Telomeric repeats are required for early S-phase origin firing at a DSB A ChIP analysis of Polε binding to a DSB at the *adh4* locus, devoid of TG-repeat sequences. The same strain was either induced (right) or not (left) for HO cleavage by using glucose or galactose during G1 arrest, respectively.B Similar analysis to (A) but in a strain where the HO cut was made at an internal, and late replicating, locus near *ARS1412*. This DSB was also lacking adjacent TG-repeats.C Similar analysis to (B) but the internal DSB had a short TG-tract on the side of the break where *ARS1412* is located.D–F Analysis of replication intermediates by alkaline gel electrophoresis as in Fig 2B. Strain in (D) is same as in (A), (E) same as in (B) and (F) same as in (C).

The observation that DSBs flanked by short (but not long) arrays of telomeric repeats caused a local change in the timing of origin firing raised the question of whether DSBs lacking telomeric repeats altogether might also lead to such a change. In striking contrast to what was seen at the *adh4* locus with the short-TG DSB, in the absence of telomeric repeats we failed to detect early S Polε association with the DSB (Fig 5A). This was confirmed at a second TG-less DSB, at the late-replicating *ARS1412* locus, about 200 kb from the left telomere of chromosome *XIV* (Fig 5B). Importantly, the insertion of a short array of telomeric repeats was able to induce the early recruitment of Polε also at this internal locus (Fig 5C). Analysis of replication intermediates for these strains confirmed that origin firing in presence of HU took place at the DSB only in the strain with TG-repeats at the break site, at *adh4* (Fig 2B, top left) or *ARS1412* (Fig 5F, left), but not in strains where the DSB was devoid of TG-repeats, either at *adh4* (Fig 5D, left) or *ARS1412* (Fig 5E, left). The failure to observe replication intermediates in the TG-less DSBs was not caused by loss of DNA to unchecked resection (Supplementary Fig S3). These results indicate that the shift in the timing of origin firing at DNA breaks is not a general phenomenon but is instead specific for those breaks that are flanked by telomeric repeats, suggesting that this is a unique characteristic of telomeric loci. Conceivably, this might reflect a specific role of Tel1 over Mec1 in the process [15,27,28] or be due to specific characteristics of the chromatin environment at telomeres. We have failed to observe an effect of enzymatically inactive Sir2 histone deacetylase and Tel1/Mec1-dependent phosphorylation of histone H2A on the replication timing of a short-TG DSB (Supplementary Fig S4).

## Conclusions

Our work identifies a novel function for Tel1 in modulating the timing of origin firing specifically at telomeres. Recent evidence has revealed that some negative regulators of telomerase (Taz1 in fission yeast; Rif1 and Rif2 in budding yeast) act at least in part by restricting the action of telomerase within the cell cycle [29–31]. We suggest that regulation of replication timing by Tel1 adds another layer to the regulation of telomerase at endogenous telomeres. These findings extend the recognised role of Tel1 in enhancing the action of telomerase at telomeres and demonstrate that this kinase controls not only the extent but also the timing of telomerase telomere association. It will be interesting to determine whether this function of Tel1 in controlling origin activity might be related to the role of ATR in promoting the activation of dormant origins at sites of replication stress in higher eukaryotes. Because the effect of Tel1 is confined to telomeres, it is tempting to speculate that whereas the DDK kinase appears primarily to act directly on replicative factors, Tel1 might act to modify the chromatin context at telomeric origins.

## Materials and Methods

### Strains and plasmids

All strains were generated in the W303 background (*MATa ade2-1 his3-11,15 leu2-3,112 trp1-1 ura3-1 can1-100 RAD5*). A list of the strains used, including those in Supplementary Figures, is reported in Supplementary Table S1. The plasmids used to modify the *ADH4*, *NAR1* and *YER188W* loci, at chromosomes *VII*, *XIV* and *V,* respectively, are listed in Supplementary Table S2. Standard budding yeast handling and growth conditions were used. Rich medium was YPAD, and drop-out media were made using pre-made mixes from USB.

### Induction of HO endonuclease and synchronisation of yeast cultures

To analyse cells in S phase, cells were grown in overnight cultures in the appropriate drop-out SC medium containing raffinose at 30°C. The cultures were diluted into YPA-raffinose and arrested in G1 phase of the cell cycle with 0.025 μM α-factor. Cells were then switched to YPA-galactose for 4 h at 30°C, while maintaining the arrest with 0.025 μM α-factor. Cells were released into S phase in YPAD containing 0.125 mg/ml pronase at 18°C. For alkaline smear analysis, cells were instead released from G1 arrest into S phase in the presence of 200 mM hydroxyurea.

To analyse Cdc45 recruitment in G1, overnight cultures in the appropriate drop-out SC medium containing raffinose were diluted into YPA-raffinose and grown at 30°C until in log phase. Cells were then arrested in G2/M phase of the cell cycle with the addition of 20 μg/ml nocodazole to the media and incubating the cells for 90 min at 30°C. Cells were then washed and released into YPA-galactose and incubated with 0.025 μM α-factor for 4 h at 30°C.

### Analysis of replication intermediates

Analysis of DNA replication intermediates was performed by 2D gel electrophoresis. DNA was prepared using Qiagen genomic prep columns and the DNA was digested with either XmnI (to analyse 5.2 kb *ARS700.5* fragment and 6.5 kb *ARS522* fragment) or PstI (to analyse 7.0 kb *ARS607* fragment). Probes were prepared by PCR using oligos DO958/959 (*ARS700.5*), DO1272/1279 (*ARS607)*, and DO1275/1276 (*ARS522*) (see Supplementary Table S3). For analysis of DNA replication intermediates by alkaline agarose gel electrophoresis probes used were the same as for 2D gels, except that *ARS305* was probed with a PCR product obtained with oligos DO1787/DO1788.

### ChIP

ChIP was performed as described in Supplementary Methods. Immunoprecipitations were carried out with anti-Myc 9E10 (supernatant from a 9E10 hybridoma cell-line) against C-terminally Myc-tagged proteins or with anti-Flag antibody (Sigma M2 antibody, F3165) against C-terminally Flag-tagged proteins and ProteinG Dynabeads (Invitrogen). Quantitation of immunoprecipitated DNA was obtained by real-time PCR using SYBR Green detection on a Roche Light Cycler 480 II instrument and expressed as per cent of starting (input) material. Primers used are listed in Supplementary Table S3.

## Supporting Information

Supplementary Information

Review Process File
