# Supplementary figures and images for: Tel1ATM dictates the replication timing of short yeast telomeres
Source: EMBO Rep. 2014 Aug 13;15(10):1093–101. doi: 10.15252/embr.201439242 (PMC4253850; doi:10.15252/embr.201439242)

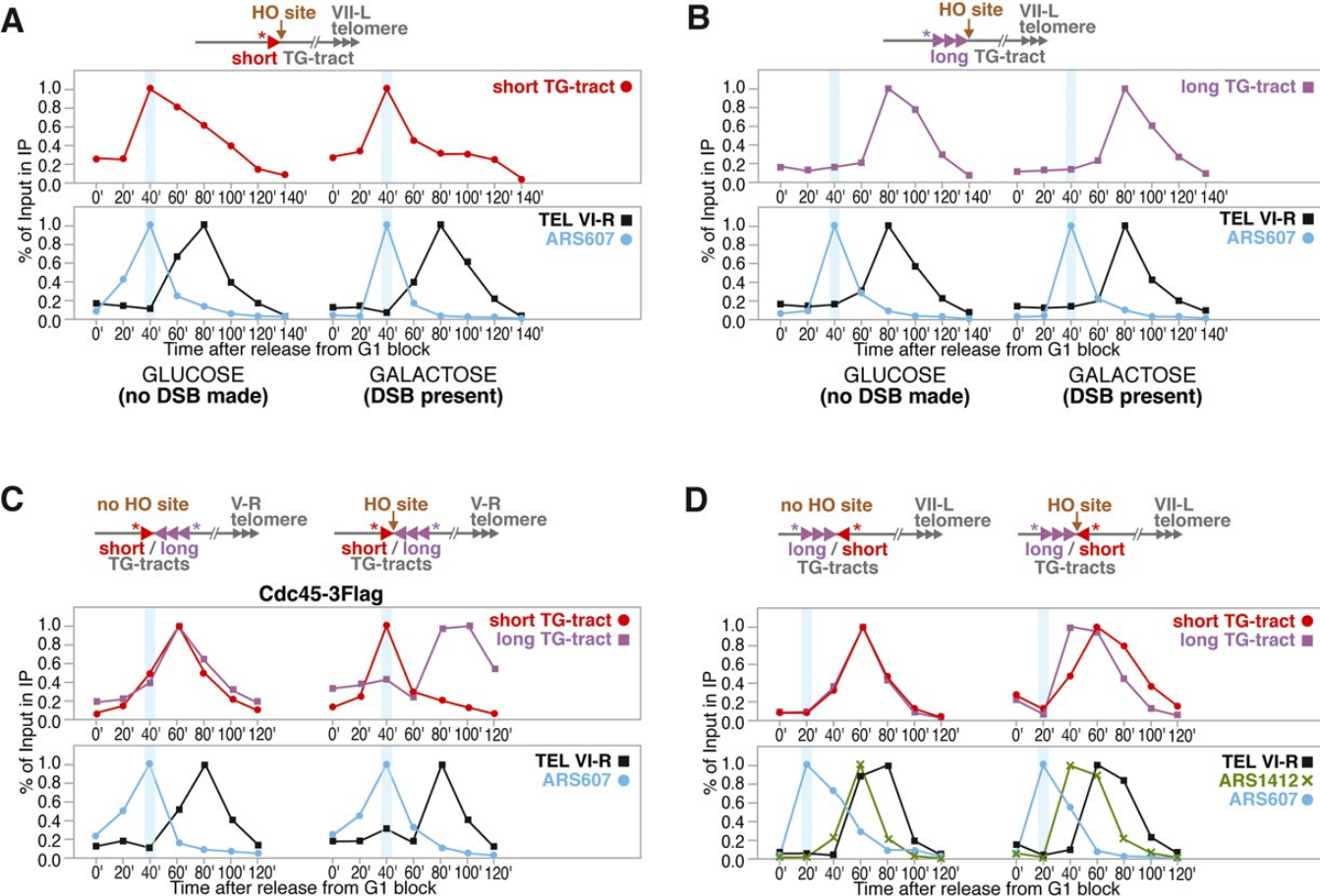

**Figure S1.** Replication timing at a DSB flanked by telomeric tracts.

Supplement: Supplementary file 1 — Supplementary Figure S1 [file embr0015-1093-sd1.pdf]

**A**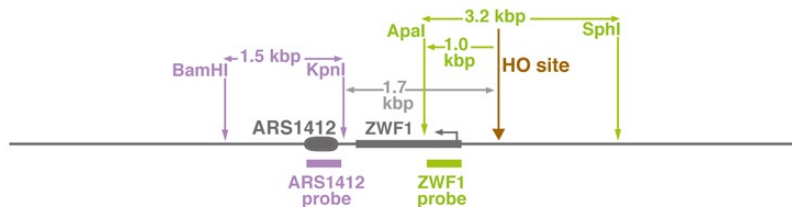**B**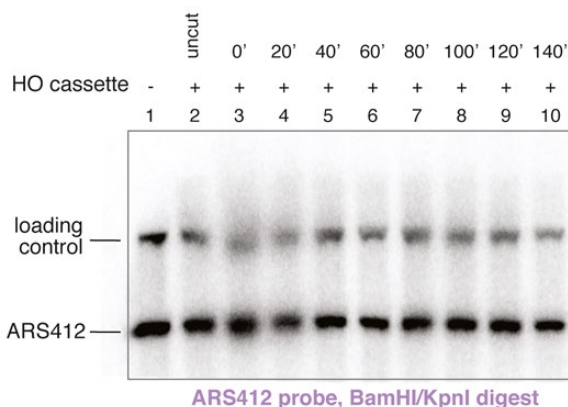**C**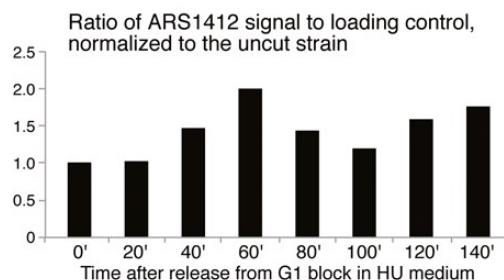**D**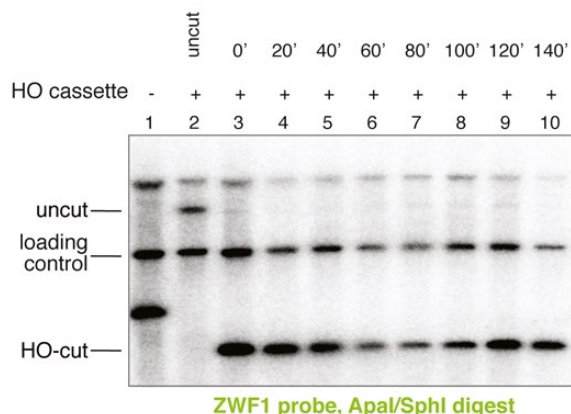**E**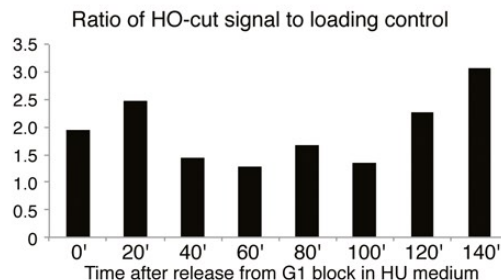

**Figure S3.** Analysis of resection at the TG-less DSB upon release into 200 mM HU for two hours.

Supplement: Supplementary file 3 — Supplementary Figure S3 [file embr0015-1093-sd3.pdf]
