# Supplementary material for: Tel1ATM dictates the replication timing of short yeast telomeres
Source: EMBO Rep. 2014 Aug 13;15(10):1093–101. doi: 10.15252/embr.201439242 (PMC4253850; doi:10.15252/embr.201439242)
Supplement: Supplementary file 2 — Supplementary Figure S2 [file embr0015-1093-sd2.pdf]

A

Mcm4 wild-type MSQQSSSPTKEDNNSSSPVVPNPDSVPPQLSSPALFYSSSSSQGDIYGRNNSQNLSQGEGNIRAAIGSSPLNFPSSSQRQNSDVFQSQG

Mcm4-6A mutant MAQQSSSPTKEDNNSSSPVVPNPDSVPPQLSSPALFYSSSSAQGDIYGRNNAQNLAQGEGNIRAAIGSSPLNFPSSAQRQNSDVFQAQG

Mcm6 wild-type MSSPFPADTPSSNRPSNSSPPPSSIGAGFGSSSGLDSQIGSRLHFPSSSQPHVSNSQTGPFVNDSTQFSSQRLQTDGSATNDMEGNEPA

Mcm6-5A mutant MSSPFPADTPSSNRPSNSSPPPSSIGAGFGSSSGLDAQIGSRLHFPSSAQPHVSNAQTGPFVNDSAQFSAQRLQTDGSATNDMEGNEPA

B

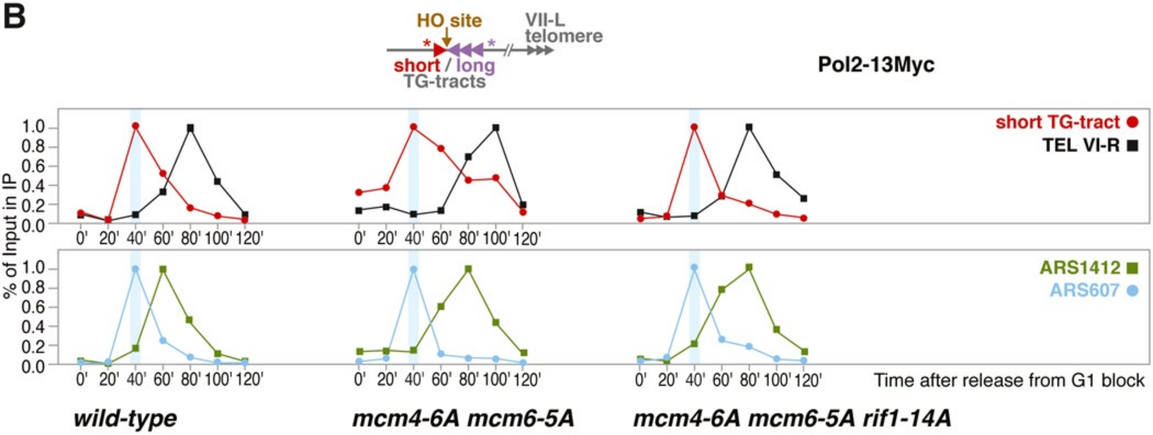

Figure S2. Phosphorylation of the N-terminal domain of Mcm4 and Mcm6 by Tel1 is not required for early Pol2 association at the DSB flanked by the short TG-tract
