# Supplementary material for: Tel1ATM dictates the replication timing of short yeast telomeres
Source: EMBO Rep. 2014 Aug 13;15(10):1093–101. doi: 10.15252/embr.201439242 (PMC4253850; doi:10.15252/embr.201439242)
Supplement: Supplementary file 4 — Supplementary Figure S4 [file embr0015-1093-sd4.pdf]

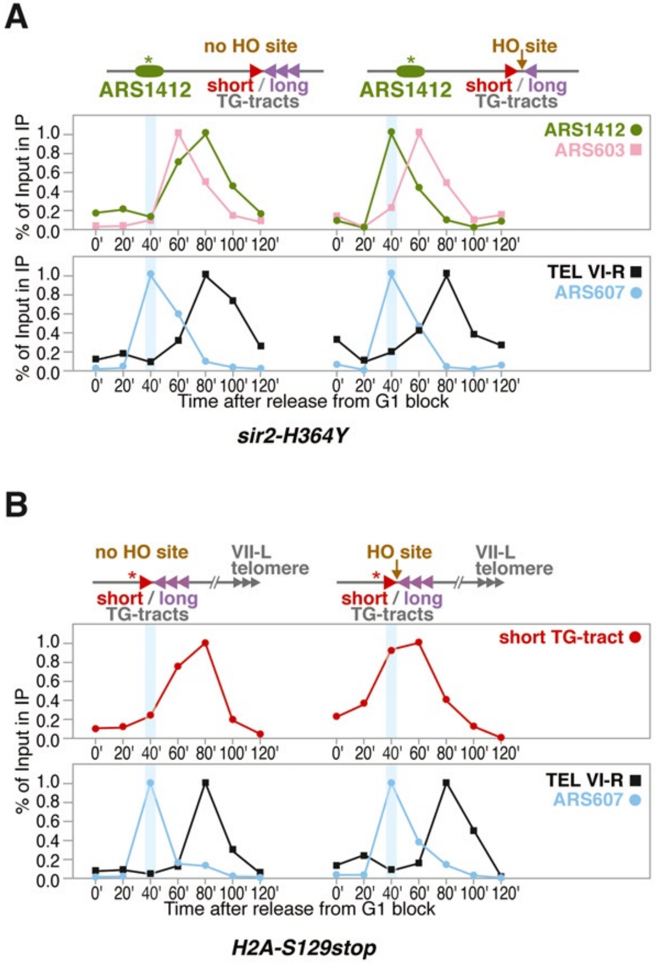


**Figure S4.** Sir2 deacetylase activity and phosphorylation of histone H2A tail are not required for the association of Polε with short-TG DSBs.
