# Supplementary material for: Tel1ATM dictates the replication timing of short yeast telomeres
Source: EMBO Rep. 2014 Aug 13;15(10):1093–101. doi: 10.15252/embr.201439242 (PMC4253850; doi:10.15252/embr.201439242)
Supplement: Supplementary file 7 — Supplementary Table S3 [file embr0015-1093-sd7.pdf]

Table S3. List of oligonucleotides used.

Primer	Amplicon	Target	Primer Sequence
**Primers used for Real Time PCR**			
FSB11a (DO1135)	SBR33	TEL VI-R	CCATGACCCAGTCCTCATTT
RSB11a (DO1136)			TGGCAAGGGTAAAAACCAGT
FSB27 (DO2521)	SBR27	centromere-proximal TG-tract at DSB (ChrVII-L)	TGGTTTCTTGTCTGGTTTCTCAAC
RSB27 (DO2522)			GAATACGCTGGTTTGCATAAAGG
FSB31 (DO1251)	SBR31	mnt2 (centromere-distal to DSB)	GATGGAATGGCGAGGTTTACC
RSB31 (DO1252)			GGGCTTAGAACGAAGGAATTGA
FSB19a (DO1138)	SBR34	ARS607	CTTTAGCTGGGTTTATGGGAGGTT
RSB19 (DO2517)			TAATGCACGAGCCGAAACAA
FSB21 (DO2208)	SBR21	ARS1412	GTGCTTTTCGGGCATAACAT
RSB21 (DO2209)			ACCGGAAATTTACCCCTACG
FSB25a (DO1232)	SBR35	TEL XV-L	CCTTACCTCCCCACTCGTTAC
RSB25 (DO2518)			ATCGTGGTTCGCTGTGGTAT
FSB10 (DO2710)	SBR36	centromere-proximal TG-tract at DSB (ChrV-R)	AGTACTGGAGCCGGAGGTACAA
RSB10a (DO1134)			GCCATGGTATCTGGTTCTCC
FSB18 (DO2519)	SBR18	ARS522	AAGCAAATTGCAGAAGGTTATGAA
RSB18 (DO2520)			TTCAAGGCTCTAGCATATGAAACG
FSB30 (DO998)	SBR30	ARS603	AATCCACCACAAAGCCCTAA
RSB30 (DO999)			CGAGGGTCGAAATCATCATC
FSB16 (DO2711)	SBR32	distal TG tract at DSB (ChrV-R)	AGCAAAAATGACCCACCAATG
RSB16a (DO1137)			GCCTGGATGGTTCAGGATAA
FSBcc1a (DO1868)	SBR47	ARS700.5	ACGGTTTTCTCGTCCACATC
RSBcc1a (DO1869)			CCGAGAATAATGAAACTGGTCTG
**Other primers used**			
DO1323			AAGCTTGTATCTCCGCGTAAGACCGCCGGAGTATGCATCTTATAGCGGTTCAGCTGAAGCTTCGTACGC
DO1324			AATAGTAGCGCTACTGGAAGCACCACGTAATAGTGGAAAAGAACTGGAAAGCATAGGCCACTAGTGGATCTG
DO958			GCTactagtTTCACCAAAGAACAAGTGGTTGCC
DO959			CCTgcggccgcCGGAAAGCTATATGATATAAACTAGC
DO1272			ACGATGGAACCTTGTTCAGG
DO1279			AGGTTCGCCATAAGCTCAAA
DO1275			GTGGGATTTATCCCCGCTAC
DO1276			TCAGGCATATCTGGGGAATC
DO1787			ATCGTGTAAGCTGGGGTGAC
DO1788			GGCAAACGTCCAAAGACAAT
DO2304			CCCCCACCAACTCCTCTCTA
DO2307			GCAAAGGGACAGATGAAGGA
DO969			CGAATTTGACGGGGCCTTCACTCAT
DO972			TTGCAGCTCCCTGGCAGAGGCCAG
DO908			CGATACGACTAGATTCTTCGCACCATTG
DO909			GTCGGCGAGGGTAACGCACAATTGGTAG
