# Supplementary material for: Tel1ATM dictates the replication timing of short yeast telomeres
Source: EMBO Rep. 2014 Aug 13;15(10):1093–101. doi: 10.15252/embr.201439242 (PMC4253850; doi:10.15252/embr.201439242)
Supplement: Supplementary file 9 — Supplementary Information [file embr0015-1093-sd9.pdf]

Tel1^{ATM} dictates the replication timing of short yeast telomeres

Carol Cooley, Anoushka Davé, Mansi Garg and Alessandro Bianchi

Running title: Tel1^{ATM} specifies telomere replication timing

Keywords: DNA replication / origin firing / replication timing / Tel1 / telomeres

Genome Damage and Stability Centre

University of Sussex

Brighton BN1 9RQ

United Kingdom

Corresponding author: Alessandro Bianchi

Email: A.Bianchi@sussex.ac.uk

Methods

Strains and plasmids

All strains were generated in the W303 background (*MATa ade2-1 his3-11,15 leu2-3,112 trp1-1 ura3-1 can1-100 RAD5*). A complete list of the strains used, including those in Supplementary Figures, is reported in Table S1. The plasmids used to modify the *ADH4*, *NAR1* and *YER188W* loci, at chromosomes *VII*, *XIV* and *V* respectively, are listed in Table S2. Standard budding yeast handling and growth conditions were used. Rich medium was YPAD, and drop-out media were made using pre-made mixes from USB.

Induction of HO endonuclease and synchronisation of yeast cultures

Cells were grown in 100 ml overnight cultures in the appropriate drop-out SC medium containing 4% raffinose. The cultures were then diluted into 300 ml of YPA 4% raffinose and grown for 2 hrs with 0.025 µM α-factor to arrest the cells in G1 phase of the cell cycle. Cells at a density of 1 x 10^7 cells/ml were then switched to YPA 4 % galactose for 4 hrs at 30°C, while maintaining the arrest with 0.025 µM α-factor. Cells were released into S-phase by washing twice with water and switching the cells to YPAD containing 0.125 mg/ml pronase at 18°C. For alkaline smear analysis, cells were instead released from G1 arrest into S-phase in the presence of 200 mM hydroxyurea.

To analyse Cdc45 recruitment in G1, cells were grown in 20 ml overnight cultures in the appropriate drop-out SC medium containing 4% raffinose. In the morning, cells were diluted into YPA 4% raffinose and grown for approximately 2 hrs at 30°C until cells were at a density of 1 x 10^7 cells/ml. Cells were then arrested in G2/M phase of the cell cycle with the addition of 20 µg/ml nocodazole to the media and incubating the cells for 90 min at 30°C. Cells were then washed and released into YPA 4 % galactose and incubated with 0.025 µM α-factor for 4 hrs at 30°C.

Analysis of replication intermediates

Analysis of DNA replication intermediates was performed by 2D gel electrophoresis [1-3]. To analyse origin firing at *ARS700.5*, Qiagen genomic column-purified DNA was digested with XmnI and probed with a probe specific for the origin-containing 5.2 kb XmnI fragment (made by PCR with primers DO958/959, see Table S3); for *ARS607*, DNA was digested with PstI and the 7.0 kb fragment detected with a probe made with DO1272/1279; for *ARS522*, DNA was digested with XmnI and the 6.5 kb fragment detected with a probe made with DO1275/1276.

Analysis of DNA replication intermediates by alkaline agarose gel electrophoresis was performed as described previously [4]. Where applicable, probes used were the same as for 2D gels, and *ARS305* was probed with a PCR product obtained with oligos DO1787/DO1788, (Table S3). Briefly, DNA was prepared by lysing 1 x 10^8 cells in 2% Triton X-100, 1% SDS, 100 mM NaCl, 10 mM Tris-HCl pH 8.0, 1 mM EDTA, 0.01% 2-mercaptoethanol with lyticase (400U, USB). The DNA was then separated on a 1% denaturing alkaline gel (50 mM NaOH, 1 mM EDTA) and Southern blotting was carried out under denaturing conditions. DNA was probed with ^{32}P-labelled probe generated by PCR using oligos indicated in Table S3.

ChIP

ChIP was performed as described previously [5, 6]. Briefly, after cross-linking in 1% formaldehyde, cells were lysed and sonicated to achieve DNA fragments <500 bp. Immunoprecipitations were carried out with anti-Myc 9E10 (supernatant from a 9E10 hybridoma cell-line) against C-terminally Myc-tagged proteins or with anti-Flag antibody (Sigma M2 antibody, F3165) against C-terminally Flag-tagged proteins and ProteinG Dynabeads (Invitrogen). Both an aliquot of sonicated cleared extract (input) and the immunoprecipitated material were de-cross-linked in TE plus 1% SDS at 65°C overnight. Quantitation of immunoprecipitated DNA was obtained by real-time PCR using SYBR Green detection [7] on a Roche Light Cycler 480 II instrument and expressed as percent of starting (input) material. Primers used are listed in Table S3.

Southern blotting and resection assay

Cells were synchronised and induced as described above and DNA was prepared and digested for southern blotting using standard budding yeast techniques. The BamHI-KpnI fragment containing *ARS1412* was probed with a PCR product made with primers DO2307/DO2304. The ApaI/SacI fragment was probed with a PCR product made with primers DO972/DO969. A probe for the Fas1 genomic locus was used as a loading control and the probe was prepared using primers DO908/DO909, (primers listed in Table S3).

References

1. Liberi G, Cotta-Ramusino C, Lopes M, Sogo J, Conti C, Bensimon A, Foiani M (2006) Methods to study replication fork collapse in budding yeast. *Methods Enzymol* **409:** 442-462

2. Wu JR, Gilbert DM (1995) Rapid DNA preparation for 2D gel analysis of replication intermediates. *Nucleic Acids Res* **23:** 3997-3998

3. Friedman KL, Brewer BJ (1995) Analysis of replication intermediates by two-dimensional agarose gel electrophoresis. *Methods Enzymol* **262:** 613-627

4. Santocanale C, Diffley JF (1998) A Mec1- and Rad53-dependent checkpoint controls late-firing origins of DNA replication. *Nature* **395:** 615-618

5. Taggart AK, Teng SC, Zakian VA (2002) Est1p as a cell cycle-regulated activator of telomere-bound telomerase. *Science* **297:** 1023-1026

6. Bianchi A, Shore D (2007) Early replication of short telomeres in budding yeast. *Cell* **128:** 1051-1062

7. Pfaffl MW (2001) A new mathematical model for relative quantification in real-time RT-PCR. *Nucleic Acids Res* **29:** e45

Figure Legends

Figure S1. Replication timing at a DSB flanked by telomeric tracts.

A. A unique DSB was introduced at the *adh4* locus (about 20 kbp form the left telomere of chromosome *VII*) by induction of the HO endonuclease with galactose during the G1 arrest (right). An uncleaved control was produced by incubating the same strain in glucose, rather than galactose, in G1 (left). After arrest in the G1 phase with α-factor cells were released synchronously into the cell cycle at 18°C in glucose-containing medium by removing the α-factor. Samples were collected at the indicated times and subjected to immediate formaldehyde cross-linking for ChIP analysis of Polε-13Myc association with the indicated loci. Two loci were included in the analysis as early and late S-phase markers: *ARS607* and telomere *VI-R*, respectively. Association of Polε -13Myc with the proximal end of a DSB at the subtelomeric adh4 locus was determined. The HO site was flanked at its proximal end by about 80 bp of yeast telomeric sequence, oriented with the TG-strand running towards the break site with a 5' to 3' polarity. In this and all other ChIP experiments, the early S-phase time-point, characteristic of *ARS607* and short telomeres, was highlighted in the figures with a light blue bar. In all figures asterisks indicate the positions of PCR amplicons used for QPCR.

B. Same as in A but the length of the TG-tract at the DSB was about 250 bp. The right panel is the same as in Figure 1C.

C. ChIP analysis of Cdc45-3Flag binding to the indicated DSB at the *adh4* locus was conducted as in Figure 1A.

D. ChIP analysis of Polε-13Myc binding to the indicated DSB at the *adh4* locus was conducted as in Figure 1A but the relative position of the short and long TG tracts has been inverted (TG250-HO-CA80 cassette), so that the short-TG was on the distal end of the DSB. Cleavage at this locus released a 23 kbp long fragment, since more than 9 kb of exogenous sequence were introduced at the *MNT2* locus, including the *LYS2* gene, which we used to monitor cleavage in these strains. The putative single origin in this distal fragment is located at the X core element, which is very close to the terminal endogenous telomeric array. The simplest interpretation for the lack in early S phase Polε recruitment at the distal DSB is that the relatively long distance is not sufficient for the

short-TG DSB to affect origin firing at the X core origin, possibly also due to the latter's immediate proximity to an endogenous telomere of normal size. However, the long distance of the origin from the DSB would also be expected to impose some delay on the arrival of Polε at the break and the exact replication timing of the telomere-proximal region was not assessed in this experiment. (In this particular experiment cells released faster than normal and all replication markers were shifted by about 20 minutes.)

Figure S2. Phosphorylation of the N-terminal domain of Mcm4 and Mcm6 by Tel1 is not required for early Pol2 association at the DSB flanked by the short TG-tract.

A. N-terminal amino acid sequence of Mcm4 and Mcm6 showing the positions of Mec1 phosphorylation sites (green) with the amino acid substitutions used in the Mcm4-6A and Mcm6-5A mutants indicated in red.

B. ChIP analysis of Polε binding to the indicated DSB at the *adh4* locus in the indicated mutant backgrounds was conducted as in Figure S1A.

Figure S3. Analysis of resection at the TG-less DSB upon release into 200 mM HU for two hours.

A. Diagram indicating the modified *ARS1412* locus with the TG-less HO site. Distances in kbps and restriction enzymes used in panels B and D are indicated.

B. Southern blotting analysis of the BamHI-KpnI fragment encompassing *ARS1412*.

C. Quantification of the blot in (B). The assay demonstrates that the BamHI and KpnI sites are retained and therefore are present in double-stranded form.

D. Southern blotting analysis of the ApaI-SphI fragment overlapping the HO site.

E. Quantification of the blot in (D). The assay demonstrates that the ApaI sites is retained and therefore is present in double-stranded form.

Figure S4. Sir2 deacetylase activity and phosphorylation of histone H2A are not required for the association of Pol2 with short-TG DSBs.

A. ChIP analysis of Polε binding to the indicated DSB at the *ARS1412* locus in a *Sir2-H364Y* mutant background.

B. ChIP analysis of Polε binding to the indicated DSB at the *adh4* loci in strains carrying the *H2A-S129stop* mutation. Both experiments were conducted as described in Figure S1A.
