# Supplementary material for: Tel1ATM dictates the replication timing of short yeast telomeres
Source: EMBO Rep. 2014 Aug 13;15(10):1093–101. doi: 10.15252/embr.201439242 (PMC4253850; doi:10.15252/embr.201439242)
Supplement: Supplementary file 10 — Review Process File [file embr0015-1093-sd10.pdf]

EMBO
reports

Manuscript EMBOR-2014-39242

Tel1ATM dictates the replication timing of short yeast telomeres

Carol Cooley, Anoushka Dave, Mansi Garg and Alessandro Bianchi

Corresponding author: Alessandro Bianchi, University of Sussex

Review timeline:

Submission date:	30 June 2014
Accepted:	23 July 2014

Transaction Report:

No Peer Review Process File is available with this article, as the authors have chosen not to make the review process public in this case.

© European Molecular Biology Organization
